# Trends in financial payments from industry to US cancer centers, 2014-2021

**DOI:** 10.1093/jncics/pkae015

**Published:** 2024-06-03

**Authors:** Nirjhar Chakraborty, Meredith Brown, Sonia Persaud, Grace Gallagher, Niti U Trivedi, Peter B Bach, Aaron P Mitchell

**Affiliations:** Department of Epidemiology and Biostatistics, Memorial Sloan Kettering Cancer Center, New York, NY, USA; US Digital Corps, Office of Technology Transformation Services, US General Services Administration, Washington, DC, USA; Department of Epidemiology and Biostatistics, Memorial Sloan Kettering Cancer Center, New York, NY, USA; Department of Epidemiology and Biostatistics, Memorial Sloan Kettering Cancer Center, New York, NY, USA; Department of Epidemiology and Biostatistics, Memorial Sloan Kettering Cancer Center, New York, NY, USA; Delfi Diagnostics, Baltimore, MD, USA; Department of Epidemiology and Biostatistics, Memorial Sloan Kettering Cancer Center, New York, NY, USA; Delfi Diagnostics, Baltimore, MD, USA; Department of Epidemiology and Biostatistics, Memorial Sloan Kettering Cancer Center, New York, NY, USA

## Abstract

**Background:**

Industry payments to US cancer centers are poorly understood.

**Methods:**

US National Cancer Institute (NCI)–designated comprehensive cancer centers were identified (n = 51). Industry payments to NCI–designated comprehensive cancer centers from 2014 to 2021 were obtained from Open Payments and National Institutes of Health (NIH) grant funding from NIH Research Portfolio Online Reporting Tools (RePORT). Given our focus on cancer centers, we measured the subset of industry payments related to cancer drugs specifically and the subset of NIH funding from the NCI.

**Results:**

Despite a pandemic-related decline in 2020-2021, cancer-related industry payments to NCI–designated comprehensive cancer centers increased from $482 million in 2014 to $972 million in 2021. Over the same period, NCI research grant funding increased from $2 481  million to $2 724  million. The large majority of nonresearch payments were royalties and licensing payments.

**Conclusion:**

Industry payments to NCI–designated comprehensive cancer centers increased substantially more than NCI funding in recent years but were also more variable. These trends raise concerns regarding the influence and instability of industry payments.

Financial relationships between health-care providers and the pharmaceutical industry have long raised concerns regarding medical professionalism and patient care (1). Despite these concerns, physician-industry financial relationships remain common (2), with US physicians receiving more than $2 billion in direct, personal payments from the drug industry each year.

Empirical studies of physician-industry financial relationships have supported such concerns. Multiple studies have identified a consistent association between physicians’ receipt of industry payments and their prescribing practices (3). Several studies have applied causal inference methods, supporting a causal impact of payments on physician behavior (4,5). In some cases, industry payments have been associated with low-value or even harmful prescribing (6-8).

Despite this large body of recent research on industry payments to physicians, payments to teaching hospitals have received relatively little attention. Industry payments to hospitals have the potential to cause institutional conflict of interests, which may threaten the integrity of clinical research and the public mission of health-care institutions (9,10). US cancer centers receive federal funding through the National Cancer Institute (NCI) to support their public missions but also to conduct a large amount of industry-funded clinical research. Funding for clinical research has shifted toward industry in recent decades (11), potentially affecting the revenue sources of cancer centers. Therefore, we aimed to assess the distribution and trends in industry payments and public (NIH) funding to US cancer centers.

## Methods

The study sample included all NCI–designated comprehensive cancer centers, except St Jude Children’s Research Hospital because of its exclusive focus on pediatric patients. Industry payment data were obtained from Open Payments, and National Institutes of Health (NIH) funding from Research Portfolio Online Reporting Tools. Because Open Payments typically does not report industry payments to cancer centers separately from their parent teaching hospitals or medical centers and the National Institutes of Health (NIH) typically does not report grant payments to hospitals or medical centers separately from their parent universities, we elected to measure both categories broadly, including industry payments and NIH grant payments to all hospitals within the contiguous provider network affiliated with each NCI–designated comprehensive cancer centers.

To measure industry payments, we first identified all teaching hospitals and nonteaching hospitals affiliated with each NCI–designated comprehensive cancer center (details in Supplementary Methods, available online). We then summed research payments and general payments across all such entities for each NCI–designated comprehensive cancer center. To measure NIH funding, we included all funding mechanisms, including research project grants, center grants, training grants, and contracts. Similar to industry payments, NIH grants across all affiliated hospitals were attributed to the NCI–designated comprehensive cancer centers.

We tabulated NIH funding and industry payments by calendar year, in aggregate and within individual NCI–designated comprehensive cancer centers. Because our motivating interest was cancer centers, our primary analysis focused on the subset of NIH grants from the NCI and the subset of industry payments related to cancer drugs (identified by manual review of the covered product class and name fields within Open Payments). However, because even these narrower subsets would likely capture some activity in the broader medical center or university—outside of the cancer center—we also conducted a separate analysis of freestanding cancer hospitals (identified as the union of Prospective Payment System-exempt cancer hospitals and NCI–designated comprehensive cancer centers), as these are typically treated as separate entities within NIH and Open Payments data. All values were inflation adjusted to 2021 US dollars using the Consumer Price Index for medical care from the US Bureau of Labor Statistics.

## Results

A total of 51 NCI–designated comprehensive cancer centers were in our cohort. Across all NCI–designated comprehensive cancer centers, industry payments increased from $1 811 million in 2014 to a peak of $3 015 million in 2019 before declining to $1 977 million in 2021 (Supplementary Table 1, available online). The proportion of industry payments that were general (as opposed to research) payments ranged from 29% in 2014 to a peak of 39% in 2019 before declining to 13% in 2021. The large majority (97%, not shown) of general payments were royalty or license payments.

**Figure 1.**
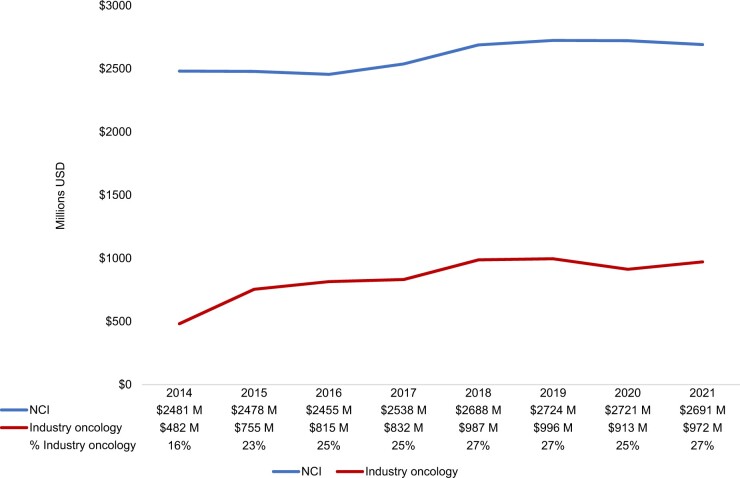
Payments received by National Cancer Institute (NCI)–designated comprehensive cancer centers from industry and NCI sources, 2014-2021. NCI payments include all funding mechanisms, including research project grants, center grants, training grants, and contracts. Industry payments include general and research payments and are shown in total (industry total) and in the subset related to oncology drugs (industry oncology). The upward trend in 2019 is accounted for in large part by a single $300 000 000 payment from Takeda Pharmaceuticals to MD Anderson (payment type: royalty or license). 2021 USD. M = million; USD = US dollars.

**Figure 2.**
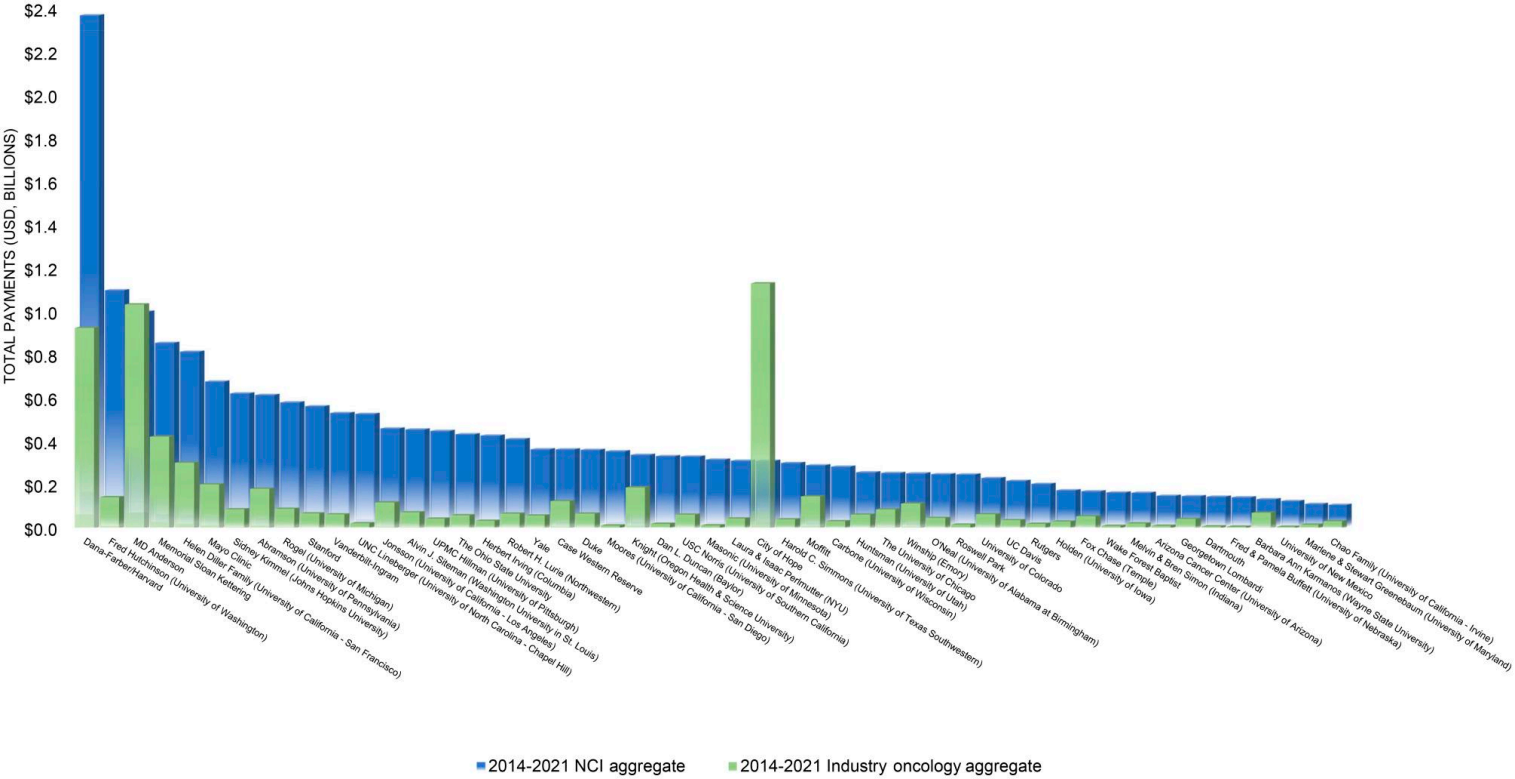
National Cancer Institute (NCI) and industry payments received by NCI–designated Comprehensive Cancer Centers, 2014-2021. NCI payments include all funding mechanisms, including research project grants, center grants, training grants, and contracts. Industry payments include general and research payments. 2021 USD. USD = US dollars.

Oncology-related industry payments increased substantially during the study period, from $482 million in 2014 to $972 million in 2021 (Figure 1). NCI funding increased slightly, from $2 481 million  to $2 691  million. Given these trends, the portion of all oncology-related funding (industry and NCI) comprised of industry payments increased from 16% in 2014 to 27% in 2021. NCI funding exceeded oncology-related industry payments at the large majority of NCI–designated comprehensive cancer centers (Figure 2). Oncology-related industry payments exceeded NCI funding at 2 NCI–designated comprehensive cancer centers: City of Hope ($1 137  million vs $311  million) and MD Anderson ($1 040 million vs $1 001 million) (Supplementary Table 2, available online). City of Hope was the only NCI–designated comprehensive cancer center for which overall industry payments exceeded NIH funding (not shown). Within the subset of Prospective Payment System-exempt cancer hospitals, aggregate industry payments exceeded NCI funding for the duration of the study period (not shown). Industry payments increased from $1 228  million in 2014 to $2 416 million in 2019 then declined to $1 347  million in 2021, while NCI funding remained relatively stable.

## Discussion

Despite a pandemic-related decline (12), industry payments to NCI–designated comprehensive cancer centers increased overall from 2014 to 2021, reflecting the well documented trend toward industry-funded cancer clinical research (13). Though public funding remains a substantially greater proportion of overall NCI–designated comprehensive cancer center revenue, oncology-related industry payments increased more quickly than NCI funding, especially during the prepandemic period, narrowing the gap.

Industry payments to cancer centers have many benefits. The predominance of royalty and licensing payments (among general payments) suggests NCI–designated comprehensive cancer centers derive substantial revenue from technologies developed with industry collaboration. Other less common general payment categories include grants, education, and charitable contributions, which may support provider training on new drugs and devices or discretionary research budgets. Research payments are made pursuant to clinical research activities, which aim to develop new cancer therapies. However, industry payments may also have adverse consequences. Industry-funded education is often biased (14,15). Industry research funding is central to the concept of institutional conflict of interest (9). A reliance on industry-derived revenue may limit an institution’s investigators in their ability to dispassionately evaluate and prioritize the paying company’s products and research interests. As industry payments increase as a share of cancer center revenue, concerns regarding potential institutional conflict of interest are not unwarranted.

The substantial pandemic-related decline in industry payments from 2019 to 2020 highlights another potential concern—their instability. Although public grant funding remained nearly unchanged, industry payments may be more reactive to economic conditions in the short term, reducing this revenue stream at a time when cancer centers were already struggling because of low patient volumes.

The variation in industry payments among NCI–designated comprehensive cancer centers likely reflects underlying variation in participation in industry-sponsored research and licensing agreements. The 2 largest outlier NCI–designated comprehensive cancer centers in terms of industry payments, City of Hope and MD Anderson, received substantial licensing revenue from Genentech and Takeda, respectively.

Our study is limited in its focus on industry payments and NIH grants, and other sources of NCI–designated comprehensive cancer center revenue (eg, philanthropic, clinical) were omitted. Because industry payments and NIH grants to NCI–designated comprehensive cancer centers are often not reported separately from the centers’ parent hospital or university, our results also contain monies paid to these broader organizations that were not directly cancer center related. However, the similar trends within the Prospective Payment System-exempt subset, which are reported separately, suggest that this consideration did not drive results. Finally, overall totals for NIH funding and industry payments may disproportionately reflect trends within outlier centers; Harvard Cancer Center accounted for more than 10% of aggregate NCI funding across all NCI–designated comprehensive cancer centers, and City of Hope for more than 15% of aggregate oncology-related industry payments.

## Supplementary Material

pkae015_Supplementary_Data

## Data Availability

All data sources used in this study are available for free download to the public.
