# Supplementary material for: Trends in financial payments from industry to US cancer centers, 2014-2021
Source: JNCI Cancer Spectr. 2024 Jun 3;8(3):pkae015. doi: 10.1093/jncics/pkae015 (PMC11144522; doi:10.1093/jncics/pkae015)
Supplement: pkae015_Supplementary_Data [file pkae015_supplementary_data.pdf]

Supplementary Material

Trends in Financial Payments from Industry to US Cancer Centers, 2014-2021

Nirjhar Chakraborty, MPH,[1] Meredith Brown,[2*] Sonia Persaud, MPH,[1] Grace Gallagher,[1] Niti U. Trivedi, MPH,[3] Peter B. Bach, MD, MAPP,[3] and Aaron P. Mitchell, MD, MPH[1]

1. Memorial Sloan Kettering Cancer Center, Department of Epidemiology and Biostatistics

2. US Digital Corps, Department of Commerce

3. Delfi Diagnostics

	Page
Supplementary Methods	2
Supplement Citations	3
Supplementary Table 1: Industry payments to NCI-designated cancer centers, 2014-2021	4
Supplementary Table 2. NCI funding and oncology drug-related industry payments at each NCI-designated comprehensive cancer center (2014-2021)	5

Supplementary Methods

To attribute industry payments to cancer centers:

To measure industry payments, we first identified manually the flagship teaching hospital associated with each NCI-designated Comprehensive Cancer Center (NCICCC) within Open Payments. We then obtained and manually reviewed financial reports from each hospital (or health system, where appropriate) to identify additional affiliated teaching hospitals which would share or directly refer patients to the NCICCC, or which would be the reported recipient entity for payments made to faculty at that NCICCC (for example, a substantial amount of industry research payments to the University of California San Francisco related to clinical trials on which UCSF oncology faculty are principal investigators are received by Langley Porter Psychiatric Hospital, as opposed to the more easily identified UCSF Medical Center). Because many academic medical centers are closely related to entities which are not teaching hospitals as defined under Open Payments, but which still receive substantial reported industry payments, we also included payments to these "non-covered" recipient entities (NCREs) (for example, in many cases affiliated university systems appear in Open Payments as NCREs). We accomplished this by identifying a common set of research faculty: we first identified the set of physicians who were Principal Investigator on any Research Payments (RP) to each NCICCC's constituent teaching hospitals during the study period, then identified all RP on which these physicians were PI, then the full set of NCREs (a distinct Open Payments field)[1] for these RP, and manually reviewed these NCREs for inclusion. To calculate industry payments associated with each NCICCC, we summed General Payments (GP) and RP across all teaching hospitals and NCREs thus identified.

To identify oncology-related industry payments:

1) Across all observed industry payments in the dataset, we identified the unique set of product categories (N = 1,909), manually reviewed them, and included all those clearly related to cancer (eg., 'CANCER,' 'Oncology,') within our definition of oncology-related payment.

2) We identified all additional product categories that were potentially related to cancer (eg., 'cellular therapy,' 'Hematology,' 'Immunotherapy'). For each of these categories, we then identified the set of unique drug names which were ever paired with one of these categories (N = 118). We manually reviewed this set of drug names for any antineoplastic or supportive care agents, and included them within our definition of oncology-related payment.

Supplement Citations

1. Open Payments Frequently Asked Questions. Published online July 14, 2023. Accessed September 1, 2023. https://www.cms.gov/OpenPayments/Downloads/open-payments-general-faq.pdf

Supplementary Table 1: Industry payments to NCI-designated cancer centers, 2014-2021. Payments are summed across all cancer centers. Industry Total reflects the sum of the General Payments and Research Payments categories; the percentages in the General Payments and Research Payments rows reflect the percentage of Industry Total. General and Research payments are also shown in terms of subset of payments that were related to oncology drugs vs. not related to oncology drugs. Research Payments are also shown in terms of the subset of payments directed towards covered recipients (eg., teaching hospitals) vs. non-covered recipients (eg., non-teaching hospitals, universities). Totals may not reflect the exact sum of their components due to rounding. 2021 USD.

	**2014**		**2015**		**2016**		**2017**		**2018**		**2019**		**2020**		**2021**	
**Industry Total**	**$1,810,970,721**		**$2,060,372,259**		**$2,285,504,484**		**$2,198,964,268**		**$2,440,257,454**		**$3,014,857,121**		**$2,080,876,187**		**$1,976,661,268**	
**General Payments**	$527,151,592	29%	$587,589,811	29%	$654,202,828	29%	$624,461,958	28%	$753,386,386	31%	$1,173,494,982	39%	$368,369,211	18%	$261,513,093	13%
Non-oncology	$365,575,122	69%	$414,148,030	70%	$479,160,330	73%	$468,398,920	75%	$572,034,606	76%	$977,009,425	83%	$223,567,375	61%	$186,746,484	71%
Oncology	$161,576,469	31%	$173,441,781	30%	$175,042,498	27%	$156,063,037	25%	$181,351,780	24%	$196,485,557	17%	$144,801,836	39%	$74,766,609	29%
**Research Payments**	$1,283,819,129	71%	$1,472,782,448	71%	$1,631,301,656	71%	$1,574,502,311	72%	$1,686,871,068	69%	$1,841,362,139	61%	$1,712,506,976	82%	$1,715,148,176	87%
Non-oncology	$963,258,903	75%	$891,181,273	61%	$991,191,213	61%	$899,025,405	57%	$881,191,889	52%	$1,041,871,548	57%	$944,390,996	55%	$818,289,852	48%
Covered	$637,252,263	66%	$545,138,692	61%	$564,085,270	57%	$555,967,247	62%	$569,634,464	65%	$669,846,809	64%	$625,611,629	66%	$535,079,165	65%
Non-Covered	$326,006,641	34%	$346,042,581	39%	$427,105,943	43%	$343,058,158	38%	$311,557,425	35%	$372,024,739	36%	$318,779,367	34%	$283,210,687	35%
Oncology	$320,560,226	25%	$581,601,175	39%	$640,110,443	39%	$675,476,906	43%	$805,679,179	48%	$799,490,591	43%	$768,115,980	45%	$896,858,324	52%
Covered	$167,798,867	52%	$207,518,009	36%	$322,832,136	50%	$342,712,404	51%	$428,053,737	53%	$479,080,394	60%	$480,938,268	63%	$590,814,128	66%
Non-Covered	$152,761,360	48%	$374,083,166	64%	$317,278,306	50%	$332,764,502	49%	$377,625,442	47%	$320,410,196	40%	$287,177,712	37%	$306,044,196	34%

Supplementary Table 2: NCI funding and oncology drug-related industry payments from 2014-2021, by NCI Comprehensive Cancer Center. For each NCI Comprehensive Cancer Center, total grant funding from the NCI (NCI) and industry payments (both general and research) related to cancer drugs (Industry Oncology) are shown, as well as the percentage of the total of both payment sources constituted by industry (% Industry Oncology). 2021 USD.

NCICCC	Payment Source	2014	2015	2016	2017	2018	2019	2020	2021
Dan L Duncan Comprehensive Cancer Center (Baylor)	*NCI*	$38,311,395	$38,082,433	$35,989,836	$41,349,101	$45,298,770	$41,036,946	$45,013,853	$47,489,745
	Industry Oncology	$1,102,135	$2,375,524	$4,221,125	$3,961,332	$1,899,724	$2,557,286	$2,991,781	$2,224,982
	% Industry Oncology	3%	6%	10%	9%	4%	6%	6%	4%
Case Comprehensive Cancer Center (Case Western)	*NCI*	$39,637,354	$44,978,605	$44,189,319	$45,619,741	$46,125,166	$44,755,289	$51,624,646	$47,726,056
	Industry Oncology	$4,140,201	$8,681,490	$9,910,951	$22,036,339	$19,355,563	$18,346,563	$20,962,271	$24,351,040
	% Industry Oncology	9%	16%	18%	33%	30%	29%	29%	34%
City of Hope Comprehensive Cancer Center	*NCI*	$32,263,295	$32,238,681	$27,828,425	$30,016,137	$42,616,307	$46,647,929	$52,433,444	$46,556,692
	Industry Oncology	$153,896,831	$165,198,882	$170,545,611	$160,100,207	$183,941,794	$184,337,727	$86,578,443	$32,773,758
	% Industry Oncology	83%	84%	86%	84%	81%	80%	62%	41%
Dartmouth Cancer Center	*NCI*	$21,765,193	$20,568,177	$15,672,023	$21,333,067	$15,128,792	$16,072,036	$17,668,223	$14,120,458
	Industry Oncology	$1,601,875	$915,306	$1,008,969	$1,031,697	$1,852,147	$1,433,645	$565,958	$549,310
	% Industry Oncology	7%	4%	6%	5%	11%	8%	3%	4%
Duke Cancer Institute	*NCI*	$40,620,513	$41,772,643	$44,201,428	$39,880,215	$55,677,919	$51,533,450	$46,592,972	$41,950,519
	Industry Oncology	$8,084,589	$7,114,820	$5,253,125	$8,539,789	$11,459,073	$12,332,866	$7,707,023	$8,206,169
	% Industry Oncology	17%	15%	11%	18%	17%	19%	14%	16%
Winship Cancer Institute (Emory)	*NCI*	$28,922,441	$32,786,312	$27,566,239	$29,453,345	$28,266,481	$34,070,287	$37,779,598	$32,836,691
	Industry Oncology	$8,742,802	$16,361,033	$13,849,710	$13,590,509	$11,453,855	$14,472,750	$21,663,181	$16,246,032
	% Industry Oncology	23%	33%	33%	32%	29%	30%	36%	33%
Fox Chase Cancer Center (Temple)	*NCI*	$24,236,078	$21,323,219	$21,051,527	$20,446,374	$22,230,245	$20,165,196	$22,584,073	$15,734,007
	Industry Oncology	$1,453,636	$5,239,812	$8,980,520	$7,839,582	$11,369,672	$6,271,541	$6,802,114	$8,433,326
	% Industry Oncology	6%	20%	30%	28%	34%	24%	23%	35%
Fred and Pamela Buffett Cancer Center (University of Nebraska)	*NCI*	$15,724,235	$15,278,688	$14,916,275	$17,035,266	$21,191,960	$17,599,396	$17,526,493	$19,987,679
	Industry Oncology	$1,196,108	$866,046	$671,212	$318,468	$428,344	$1,588,312	$1,327,283	$1,233,034
	% Industry Oncology	7%	5%	4%	2%	2%	8%	7%	6%
Fred Hutchinson Cancer Research Center (University of Washington)	*NCI*	$142,377,679	$145,301,299	$135,288,548	$131,577,548	$130,843,559	$146,638,386	$135,827,273	$131,930,445
	Industry Oncology	$7,420,211	$11,158,835	$16,338,394	$17,673,312	$24,763,920	$23,456,346	$24,935,157	$20,466,198
	% Industry Oncology	5%	7%	11%	12%	16%	14%	16%	13%
Georgetown Lombardi Comprehensive Cancer Center (Georgetown)	*NCI*	$19,875,086	$20,258,840	$18,952,740	$17,498,564	$19,049,972	$17,299,273	$15,373,607	$16,422,504
	Industry Oncology	$775,802	$1,892,807	$8,159,327	$6,905,837	$9,143,301	$7,086,025	$5,581,030	$4,384,878
	% Industry Oncology	4%	9%	30%	28%	32%	29%	27%	21%
	NCI	$262,021,185	$268,233,709	$277,079,180	$300,734,374	$333,282,972	$316,216,229	$317,490,874	$294,868,069

Dana-Farber/Harvard Cancer Center	*Industry Oncology*	$56,424,078	$77,026,222	$96,631,270	$95,331,858	$124,301,231	$134,433,980	$161,828,205	$185,800,149
	% Industry Oncology	18%	22%	26%	24%	27%	30%	34%	39%
Melvin and Bren Simon Comprehensive Cancer Center (Indiana)	*NCI*	$17,858,875	$20,931,805	$19,568,424	$20,692,209	$23,888,486	$18,563,695	$18,339,015	$20,968,902
	Industry Oncology	$1,660,761	$2,617,052	$2,812,111	$4,090,736	$3,101,965	$4,377,428	$1,941,992	$2,266,914
	% Industry Oncology	9%	11%	13%	17%	11%	19%	10%	10%
Sidney Kimmel Comprehensive Cancer Center (Johns Hopkins)	*NCI*	$84,131,027	$75,895,319	$80,675,362	$71,197,769	$76,898,282	$73,342,031	$88,713,735	$72,187,900
	Industry Oncology	$4,662,587	$7,691,670	$15,511,101	$8,961,637	$13,714,613	$12,283,465	$10,218,624	$16,329,536
	% Industry Oncology	5%	9%	16%	11%	15%	14%	10%	18%
Knight Cancer Institute (Oregon Health & Science University)	*NCI*	$44,870,551	$37,678,937	$27,810,470	$37,745,188	$41,496,503	$49,932,908	$50,198,136	$48,973,513
	Industry Oncology	$5,366,133	$99,078,839	$14,545,831	$10,638,836	$12,655,907	$16,297,853	$18,688,449	$14,640,282
	% Industry Oncology	11%	72%	34%	22%	23%	25%	27%	23%
Mayo Clinic Cancer Center	*NCI*	$94,012,624	$94,933,004	$91,360,653	$91,208,927	$95,745,372	$81,106,363	$62,280,481	$67,339,121
	Industry Oncology	$15,478,309	$30,976,444	$25,468,518	$27,701,428	$27,342,244	$28,574,310	$20,772,543	$29,626,272
	% Industry Oncology	14%	25%	22%	23%	22%	26%	25%	31%
MD Anderson Cancer Center	*NCI*	$123,700,773	$105,322,018	$113,046,402	$134,717,045	$119,572,538	$138,422,303	$136,296,395	$129,921,502
	Industry Oncology	$70,189,377	$91,804,420	$95,529,420	$112,018,797	$128,528,757	$162,595,317	$163,177,675	$216,617,802
	% Industry Oncology	36%	47%	46%	45%	52%	54%	54%	63%
Memorial Sloan Kettering Cancer Center	*NCI*	$87,579,256	$84,066,057	$96,515,275	$105,229,589	$110,330,978	$118,402,747	$126,005,780	$128,425,418
	Industry Oncology	$24,855,483	$25,018,198	$34,853,191	$43,916,411	$73,276,604	$65,642,882	$79,912,464	$81,978,911
	% Industry Oncology	22%	23%	27%	29%	40%	36%	39%	39%
Moffitt Cancer Center	*NCI*	$35,602,162	$33,813,964	$35,643,338	$33,939,178	$33,058,332	$36,360,848	$37,124,803	$44,198,324
	Industry Oncology	$7,416,403	$11,614,248	$13,407,563	$16,899,624	$26,962,683	$22,885,219	$20,467,292	$30,160,837
	% Industry Oncology	17%	26%	27%	33%	45%	39%	36%	41%
Herbert Irving Comprehensive Cancer Center (Columbia)	*NCI*	$46,917,823	$49,999,624	$48,623,545	$49,111,440	$58,135,456	$54,052,799	$56,554,111	$64,859,039
	Industry Oncology	$2,745,624	$2,922,264	$6,038,563	$9,040,818	$9,500,234	$6,407,410	$4,243,607	$5,329,829
	% Industry Oncology	6%	6%	11%	16%	14%	11%	7%	8%
Laura and Isaac Perlmutter Cancer Center (NYU)	*NCI*	$34,949,409	$32,406,280	$31,776,842	$35,080,780	$35,955,633	$43,248,525	$48,973,495	$48,893,209
	Industry Oncology	$861,450	$2,500,635	$3,962,471	$2,981,450	$5,410,141	$5,754,745	$6,144,384	$7,825,198
	% Industry Oncology	2%	7%	11%	8%	13%	12%	11%	14%
Robert H. Lurie Comprehensive Cancer Center (Northwestern)	*NCI*	$41,167,464	$50,092,167	$52,739,870	$50,918,954	$61,332,993	$52,145,887	$50,005,016	$54,028,967
	Industry Oncology	$3,564,435	$3,983,432	$10,132,350	$20,582,520	$11,896,577	$8,238,832	$5,896,557	$4,905,886
	% Industry Oncology	8%	7%	16%	29%	16%	14%	11%	8%
Roswell Park Comprehensive Cancer Center	*NCI*	$25,817,206	$29,666,377	$26,837,699	$22,889,707	$36,144,448	$35,625,719	$33,521,920	$36,149,793
	Industry Oncology	$2,674,936	$1,676,454	$3,412,601	$1,926,126	$3,276,208	$1,187,129	$2,589,549	$715,136
	% Industry Oncology	9%	5%	11%	8%	8%	3%	7%	2%
Rutgers Cancer Institute of New Jersey	*NCI*	$22,882,490	$26,263,794	$23,898,135	$21,518,199	$25,972,542	$25,050,346	$24,689,796	$31,899,487
	Industry Oncology	$295,846	$1,786,107	$3,308,815	$2,856,499	$5,419,756	$2,082,633	$2,899,382	$2,728,803

	% Industry Oncology	1%	6%	12%	12%	17%	8%	11%	8%
Stanford Cancer Institute	*NCI*	$66,178,292	$66,882,945	$63,406,186	$81,597,987	$77,234,890	$67,302,457	$65,557,498	$75,640,906
	Industry Oncology	$5,702,792	$7,540,484	$9,327,040	$8,682,718	$7,935,672	$10,725,783	$11,898,032	$8,173,674
	% Industry Oncology	8%	10%	13%	10%	9%	14%	15%	10%
The Ohio State University Comprehensive Cancer Center	*NCI*	$59,872,263	$64,760,453	$65,132,237	$56,857,808	$43,635,163	$43,943,084	$53,439,045	$46,809,062
	Industry Oncology	$2,670,975	$4,066,125	$11,510,400	$9,435,683	$8,954,896	$5,185,246	$8,784,659	$10,336,253
	% Industry Oncology	4%	6%	15%	14%	17%	11%	14%	18%
O'Neal Comprehensive Cancer Center (University of Alabama at Birmingham)	*NCI*	$30,468,396	$26,826,171	$25,040,568	$26,985,900	$30,908,595	$37,410,169	$37,438,804	$32,987,101
	Industry Oncology	$1,816,594	$7,460,660	$5,405,595	$6,967,146	$7,478,524	$6,779,278	$7,764,860	$4,821,764
	% Industry Oncology	6%	22%	18%	21%	19%	15%	17%	13%
Arizona Cancer Center (University of Arizona)	*NCI*	$20,628,008	$16,359,855	$17,782,422	$15,812,821	$16,915,563	$19,425,087	$20,408,317	$19,233,162
	Industry Oncology	$516,691	$678,790	$685,983	$712,515	$1,952,121	$3,024,295	$2,489,154	$1,822,655
	% Industry Oncology	2%	4%	4%	4%	10%	13%	11%	9%
UC Davis Comprehensive Cancer Center	*NCI*	$22,264,243	$26,243,059	$26,022,992	$32,399,656	$34,954,432	$27,227,255	$23,931,798	$23,511,884
	Industry Oncology	$1,355,287	$2,314,270	$3,078,757	$3,642,249	$4,082,093	$4,450,593	$9,386,421	$10,303,324
	% Industry Oncology	6%	8%	11%	10%	10%	14%	28%	30%
Chao Family Comprehensive Cancer Center (University of California - Irvine)	*NCI*	$11,159,315	$9,297,236	$11,143,456	$10,749,193	$11,587,299	$14,012,243	$17,836,390	$18,515,233
	Industry Oncology	$968,343	$1,099,667	$2,083,617	$1,794,441	$4,365,890	$5,256,382	$8,785,382	$8,523,506
	% Industry Oncology	8%	11%	16%	14%	27%	27%	33%	32%
Jonsson Comprehensive Cancer Center (University of California - Los Angeles)	*NCI*	$54,112,341	$55,709,248	$64,286,546	$64,644,174	$67,931,247	$59,912,383	$48,572,583	$46,060,198
	Industry Oncology	$6,317,140	$14,189,961	$16,383,311	$12,711,351	$15,150,530	$21,666,194	$17,405,789	$17,482,078
	% Industry Oncology	10%	20%	20%	16%	18%	27%	26%	28%
Moores Comprehensive Cancer Center (University of California - San Diego)	*NCI*	$40,136,337	$49,875,225	$42,263,129	$41,731,778	$42,114,496	$45,374,226	$45,889,296	$48,246,502
	Industry Oncology	$785,556	$1,714,905	$2,098,009	$2,838,537	$1,788,161	$1,357,787	$1,381,134	$1,275,693
	% Industry Oncology	2%	3%	5%	6%	4%	3%	3%	3%
Helen Diller Family Comprehensive Cancer Center (University of California - San Francisco)	*NCI*	$98,909,411	$101,335,727	$100,239,320	$104,990,736	$103,011,945	$106,761,170	$95,610,838	$104,867,208
	Industry Oncology	$10,901,358	$10,519,094	$29,071,748	$58,008,641	$78,441,215	$52,295,541	$27,901,195	$40,773,664
	% Industry Oncology	10%	9%	22%	36%	43%	33%	23%	28%
The University of Chicago Comprehensive Cancer Center	*NCI*	$27,514,041	$29,441,645	$23,740,274	$25,984,778	$26,337,977	$43,203,787	$38,393,752	$38,934,656
	Industry Oncology	$5,702,584	$8,272,454	$7,026,541	$7,542,751	$10,816,547	$21,237,330	$12,400,799	$15,808,186
	% Industry Oncology	17%	22%	23%	22%	29%	33%	24%	29%
University of Colorado Cancer Center	*NCI*	$28,959,162	$26,906,486	$27,968,289	$26,390,488	$29,831,417	$29,606,564	$30,465,529	$29,631,196
	Industry Oncology	$4,976,598	$3,745,745	$5,601,863	$6,766,844	$12,638,993	$11,649,707	$10,438,735	$10,187,233
	% Industry Oncology	15%	12%	17%	20%	30%	28%	26%	26%
Holden Comprehensive Cancer Center (University of Iowa)	*NCI*	$18,962,619	$19,902,589	$22,422,472	$22,039,914	$24,040,600	$23,278,605	$21,624,203	$20,275,620
	Industry Oncology	$1,294,114	$1,641,058	$3,786,418	$5,254,538	$2,966,419	$3,989,341	$4,628,022	$8,214,481
	% Industry Oncology	6%	8%	14%	19%	11%	15%	18%	29%
Marlene and Stewart Greenebaum Comprehensive	*NCI*	$11,654,097	$13,391,675	$14,527,885	$13,056,697	$13,744,632	$14,258,182	$15,151,535	$12,519,203
	Industry Oncology	$1,131,675	$1,631,156	$1,207,289	$1,421,561	$2,974,916	$3,020,299	$2,797,152	$3,169,620

Cancer Center (University of Maryland)	*% Industry Oncology*	9%	11%	8%	10%	18%	17%	16%	20%
Rogel Cancer Center (University of Michigan)	*NCI*	$64,730,515	$66,120,010	$67,994,157	$70,409,070	$78,649,345	$73,188,015	$83,730,589	$76,871,906
	Industry Oncology	$6,761,805	$9,477,289	$11,769,877	$9,472,191	$12,864,972	$9,841,622	$12,537,675	$19,229,439
	% Industry Oncology	9%	13%	15%	12%	14%	12%	13%	20%
Masonic Cancer Center (University of Minnesota)	*NCI*	$42,938,081	$38,899,425	$38,782,881	$39,681,908	$37,093,543	$39,870,721	$38,904,262	$40,432,135
	Industry Oncology	$551,955	$885,466	$1,376,691	$2,668,236	$2,120,843	$2,564,300	$2,293,844	$1,435,552
	% Industry Oncology	1%	2%	3%	6%	5%	6%	6%	3%
University of New Mexico Cancer Research and Treatment Center	*NCI*	$16,284,210	$14,999,046	$14,061,513	$16,687,261	$16,193,061	$14,174,459	$15,437,735	$14,432,953
	Industry Oncology	$105,828	$2,799,543	$950,576	$897,494	$638,079	$662,254	$382,116	$771,731
	% Industry Oncology	1%	16%	6%	5%	4%	4%	2%	5%
UNC Lineberger Comprehensive Cancer Center (University of North Carolina - Chapel Hill)	*NCI*	$69,347,800	$66,224,433	$61,496,422	$62,040,701	$65,406,834	$71,317,691	$69,323,859	$63,182,194
	Industry Oncology	$3,190,755	$3,807,665	$2,638,452	$2,967,416	$4,122,001	$2,587,239	$1,758,546	$2,788,968
	% Industry Oncology	4%	5%	4%	5%	6%	4%	2%	4%
Abramson Cancer Center (University of Pennsylvania)	*NCI*	$78,602,229	$79,117,146	$68,586,624	$74,495,956	$76,287,976	$88,984,390	$74,302,265	$75,529,885
	Industry Oncology	$11,299,757	$23,715,790	$73,586,250	$16,485,076	$15,449,987	$15,871,386	$15,671,824	$13,603,845
	% Industry Oncology	13%	23%	52%	18%	17%	15%	17%	15%
UPMC Hillman Cancer Center (University of Pittsburgh)	*NCI*	$63,389,670	$56,436,214	$62,009,817	$57,762,201	$58,818,101	$44,159,017	$49,330,160	$57,940,374
	Industry Oncology	$2,584,051	$3,185,029	$4,433,345	$8,076,903	$6,106,922	$7,785,546	$6,874,530	$6,529,848
	% Industry Oncology	4%	5%	7%	12%	9%	15%	12%	10%
USC Norris Comprehensive Cancer Center (University of Southern California)	*NCI*	$39,662,020	$44,039,069	$43,491,128	$38,995,626	$38,259,775	$42,075,452	$38,411,786	$45,787,188
	Industry Oncology	$2,790,950	$13,376,260	$4,698,649	$4,858,870	$7,953,629	$10,354,586	$10,198,775	$9,949,207
	% Industry Oncology	7%	23%	10%	11%	17%	20%	21%	18%
Harold C. Simmons Comprehensive Cancer Center (University of Texas Southwestern)	*NCI*	$27,342,122	$28,179,779	$27,612,155	$31,139,781	$38,285,785	$57,981,076	$42,968,518	$46,894,236
	Industry Oncology	$1,463,468	$4,635,320	$5,658,332	$4,668,727	$4,483,254	$3,178,855	$6,546,216	$11,529,484
	% Industry Oncology	5%	14%	17%	13%	10%	5%	13%	20%
Huntsman Cancer Institute (University of Utah)	*NCI*	$26,475,394	$20,498,228	$28,159,037	$33,043,361	$43,504,349	$32,475,776	$36,507,971	$35,880,072
	Industry Oncology	$4,997,035	$6,139,818	$5,476,958	$6,280,711	$9,396,063	$10,921,383	$11,107,454	$9,658,056
	% Industry Oncology	16%	23%	16%	16%	18%	25%	23%	21%
University of Wisconsin Carbone Cancer Center	*NCI*	$34,434,910	$33,939,645	$31,506,482	$33,207,823	$32,125,316	$33,066,442	$40,329,768	$44,581,703
	Industry Oncology	$1,747,665	$1,796,379	$2,113,833	$3,747,373	$4,673,555	$6,083,135	$5,778,936	$7,058,109
	% Industry Oncology	5%	5%	6%	10%	13%	16%	13%	14%
Vanderbilt-Ingram Cancer Center (Vanderbilt)	*NCI*	$77,655,397	$73,768,667	$67,367,949	$57,071,646	$68,144,467	$66,964,896	$61,686,855	$59,998,832
	Industry Oncology	$5,337,164	$8,309,704	$10,882,533	$9,792,033	$7,591,156	$7,360,206	$9,115,274	$6,750,267
	% Industry Oncology	6%	10%	14%	15%	10%	10%	13%	10%
Wake Forest Baptist Comprehensive Cancer Center	*NCI*	$14,341,002	$14,876,336	$19,902,639	$19,589,398	$21,311,269	$23,491,237	$25,122,260	$23,478,572
	Industry Oncology	$1,632,779	$943,199	$975,967	$2,115,011	$2,080,350	$1,938,718	$967,853	$674,462
	% Industry Oncology	10%	6%	5%	10%	9%	8%	4%	3%
	NCI	$50,388,283	$55,414,525	$47,861,118	$53,696,287	$51,494,847	$61,621,535	$67,078,832	$69,045,077
	Industry Oncology	$2,246,708	$14,130,405	$8,931,480	$13,850,069	$8,283,820	$6,808,374	$9,193,728	$11,656,790

Alvin J. Siteman Cancer Center (Washington University in St. Louis)	*% Industry Oncology*	4%	20%	16%	21%	14%	10%	12%	14%
The Barbara Ann Karmanos Cancer Institute (Wayne State University)	*NCI*	$25,091,396	$20,849,689	$17,372,006	$15,596,827	$13,454,918	$12,899,216	$13,007,319	$12,435,932
	Industry Oncology	$5,984,604	$8,188,482	$12,731,159	$11,983,703	$10,076,533	$9,512,207	$7,113,614	$7,561,037
	% Industry Oncology	19%	28%	42%	43%	43%	42%	35%	38%
Yale Cancer Center	*NCI*	$34,247,949	$35,916,337	$43,846,186	$41,703,612	$48,806,285	$52,001,049	$58,285,050	$49,959,538
	Industry Oncology	$2,692,852	$10,277,700	$7,109,519	$8,953,343	$10,589,506	$7,224,302	$9,421,129	$3,741,723
	% Industry Oncology	7%	22%	14%	18%	18%	12%	14%	7%
Total	NCI	$2,480,993,617	$2,478,102,814	$2,455,257,447	$2,537,556,106	$2,688,327,833	$2,724,274,769	$2,721,364,556	$2,691,150,568
	Industry	$482,136,696	$755,042,956	$815,152,941	$831,539,944	$987,030,959	$995,976,148	$912,917,817	$971,624,933
	% Industry Oncology	16%	23%	25%	25%	27%	27%	25%	27%
